# XZ-1 regulates cell apoptosis of gastric epithelial dysplasia via NF-κB/p53/Ki67 signaling pathway

**DOI:** 10.1042/BSR20171529

**Published:** 2018-06-12

**Authors:** Bo Xu, An-ming Zhang, Fang Li, Miao Cui, Ji Han, Qin Cao

**Affiliations:** 1 Internal medicine of TCM, Putuo Hospital, Shanghai University of Traditional Chinese Medicine, Floor 16, Building 8, No.164 Lanxi Road, Shanghai 200062, China; 2 Internal medicine of Gastroenterology, Putuo Hospital, Shanghai University of Traditional Chinese Medicine, Floor 16, Building 8, No.164 Lanxi Road, Shanghai 200062, China; 3 Internal medicine of TCM, Shanghai Putuo Traditional Chinese Medicine Hospital, Shanghai 200062, China

**Keywords:** apoptosis, GED, NF-κB/p53/Ki67, XZ-1

## Abstract

We aimed to determine the effect of ‘Xiaozeng No. 1’ (XZ-1) on cellular apoptosis changes of gastric epithelial dysplasia (GED) and to explore the underlying mechanism. Specimens taken from the pyloric area of the stomachs from rats in each group were subjected to Hematoxylin and Eosin (H&E) staining for pathological examination, TUNEL staining for apoptosis detection, and Western blot analysis for apoptosis-related proteins. The results showed that XZ-1 decreased GED incidence and enhanced gastric epithelial apoptosis. Furthermore, XZ-1 up-regulated the proapoptotic proteins including cleaved caspases (cysteine-dependent aspartate-specific protease) (-3, -8, and -9), Fas, Bax, and Bid, and facilitated the release of cytochrome *c* from mitochondria to the cytoplasm. Interestingly, XZ-1 enhanced protein expression of NF-κB p65, Ki67, and p53. Moreover, inhibition of NF-κB pathway suppressed the XZ-induced p53 expression, whereas inhibition of NF-κB or p53 pathway suppressed the XZ-induced Ki67. More importantly, inhibition of NF-κB or p53 pathway attenuated the XZ-1-mediated induction of gastric epithelial apoptosis and decline of GED incidence. Collectively, our results demonstrated that XZ-1, almost equivalent effect exerted by the positive control Retin-A, dramatically decreased GED incidence and enhanced gastric epithelial apoptosis. Meanwhile, XZ-1 activated the NF-κB/p53/Ki67-apoptosis signaling pathway, which might be one of the mechanisms whereby XZ-1 reversed GED.

## Introduction

Gastric epithelial dysplasia (GED) is an unequivocal neoplastic lesion and is generally accepted as a precursor to gastric adenocarcinomas [1,2]. It is widely known that GED represents the culmination of a chronic inflammation-metaplasia-dysplasia-carcinoma sequence progression [3]. The diagnosis of GED depends on the cytological and architectural abnormalities [1]. GED has been morphologically categorized into adenomatous (or intestinal) and foveolar (or gastric) types [1,4]. On the basis of the severity of histological abnormalities, GED has been graded either as low and high grade dysplasia or as mild, moderate, and severe dysplasia [5–7]. Patients with low grade dysplasia are usually younger than those with high grade dysplasia, whereas no difference in age was found between patients with high grade dysplasia and those with carcinoma [8]. The prevalence of GED reveals wide geographic differences, with incidence rates ranging from 0.5 to 3.8% in Western population but rates from 9.0 to 20.0% in regions such as Colombia and China [4,8,9]. The clinical significance of GED has been stressed because of the establishment of its close association with the risk of gastric cancer [1].

All *trans*-retinoic acid (Retin-A) has been shown to effectively reverse GED and thereby suppress its progression to gastric cancer [10]. Interestingly, recent studies have indicated that ‘Xiaozeng No. 1’ (XZ-1) effectively reversed GED and improved the clinical symptoms such as stomach ache, acid regurgitation, gasteremphraxis, and poor appetite [11]. XZ-1 can be prepared from conventional decoction of several kinds of Chinese medicinal materials including *Astragalus membranaceus* (24 g), *Atractylodes lancea* (12 g), processed *Rhizoma Pinelliae* (12 g), *Curcuma zedoary* (12 g), *Concha ostreae* (45 g), *Actinidia arguta (Sieb. & Zucc) Planch. ex Miq* (45 g), and fried *Licorice* (3 g) [11]. These Chinese medicinal materials described above have pharmacological effects of replenishing qi to invigorate the spleen, dissipating phlegm, and removing blood stasis; and the favorable effects of these Chinese medicinal materials were utilized by XZ-1 to improve clinical symptoms of GED [11]. However, the mechanism by which XZ-1 reversed GED has been not yet clearly established.

Apoptosis, also called as programmed cell death, plays essential roles in regulating growth, development, and tissue homeostasis [12]. Apoptosis is a critical mechanism causing cell death; failure or inhibition of apoptosis contributes to the development of some human malignancies [13]. Zhao et al. [14] has reported that the apoptosis index gradually decreased with the aggravation of GED and declined further in infiltrative carcinoma, indicating that the involvement of suppressive cellular apoptosis in the mutation from gastric mucous dysplasia to gastric carcinoma [14]. Nevertheless, there is not much literature concerning the association of XZ-1 with apoptosis changes of GED.

In the present study, N-methyl-N-nitro-N-nitrosoguanidine (MNNG) was used to establish GED rat models. The influence of XZ-1 on GED incidence and gastric epithelial apoptosis of GED model rats were evaluated. Changes in the protein expression of apoptosis-related proteins such as caspases (cysteine-dependent aspartate-specific protease) (-3, -8, and -9), cytochrome *c*, Fas, Bax, Bid were analyzed. Besides, the correlation between NF-κB/p53/Ki67 signaling pathway and cellular apoptosis was also studied.

## Materials and methods

### Establishment of rat GED models and treatment of drugs

A total of 90 male Wistar rats (age: 8-week-old; weight: 120–140 g) were purchased from Shanghai Laboratory Animal Center, Chinese Academy of Sciences (Shanghai, China). All experiments were performed in compliance with the guidelines for the Care and Use of Laboratory Animals of the National Institutes of Health. The present study is approved by the Ethics Committee of Shanghai Putuo Traditional Chinese Medicine Hospital (approval number: 2013-0055). The establishment of rat GED model was performed as previously described [10]. These rats were maintained in isolated cages at 22°C with a relative humidity of 50%. MNNG (1 g/ml in distilled water, Sigma Co., U.S.A.) was prepared in brown bottle for use as drinking water. Two milliliters of absolute alcohol was infused into the stomach of each rat at weeks 1, 3, 5, and 7. These rats were randomly assigned into six groups with 15 rats in each group, which are as follows:

1. Normal: the rats were fed without any drugs and served as blank control;
2. Vehicle: the rats were fed with MNNG for 24 weeks to induce GED and then given 2 ml saline solution by intragastric (IG) administration everyday.
3. XZ-1: the rats were fed with MNNG for 24 weeks to induce GED and then given daily XZ-1 (IG, 2 ml in water, Beijing Tong Ren Tang Co., China);
4. Retin-A: the rats were fed with MNNG for 24 weeks to induce GED and then given daily retinoic acid (IG, 40 μg/kg body weight, Shanghai No.6 Pharmaceutical Co., China);
5. XZ-1 + PDTC: the rats received the same treatment as XZ-1 group, except that these rats were co-treated with XZ-1 and ammonium pyrrolidinedithiocarbamate (PDTC) (IG, 100 mg/kg body weight, NF-κB inhibitor, Sigma, U.S.A.);
6. XZ-1 + PFT: the rats received the same treatment as XZ-1 group, except that these rats were cotreated with XZ-1 and pifithrin-α (PFT) (IG, 100 mg/kg body weight, p53 inhibitor, Sigma, U.S.A.).

Thirty-six weeks later, these rats were killed. Their stomachs were cut along the greater curvature and specimens were taken from the pyloric area. Paraffin sections were prepared and used for pathological examination, apoptosis analysis, and determination for the protein expression of apoptosis-related proteins.

### Hematoxylin and Eosin staining

Hematoxylin and Eosin (H&E) staining was performed to examine the pathological changes of gastric mucosa from rats. Briefly, stomachs were injected with formalin solution, opened along the greater curvature, and then further fixed in 4% formalin and embedded in paraffin before being cut into 6-μm thick sections. The prepared sections were stained using H&E following a routine staining procedure and examined by light microscopy.

### TUNEL staining

After the sections (4 µm) were dewaxed, proteinase K (20 µl/ml) were added for incubation at 37°C. Then Triton X-100 in 0.1% sodium citrate was added on ice and stood up. After that, the sections were incubated in 0.3% H_2_O_2_/methanol at room temperature for 30 min. Next, the sections were incubated with TUNEL (50 g, Boehringer Co., Germany) at 37°C in a humidified chamber and then stained with diaminobenzidine (DAB, Sigma, St. Louis, MO, U.S.A.). Finally, the sections were sealed and images were acquired by an Olympus microscope. The number of TUNEL positive cells (brown) was counted and compared with the total cells using ImagePro Plus 6.0 software and expressed as a percentage.

### Separation of the mitochondrial and cytosolic fractions

Rats were starved overnight and then their stomachs were cut along the greater curvature. Specimens from the pyloric area of the stomachs were cut into pieces and washed with buffer containing 225 mM mannitol, 75 mM sucrose, 0.5% BSA, 0.5 mM EGTA, and 30 mM Tris/HCl, pH 7.4. All the following procedures were carried out at 0–4°C. After that, specimens were gently homogenized in a Teflon Potter Elvehjem homogenizer. The preparation of cytosolic and mitochondrial extracts was carried out following the instructions from Cytoplasmic and Mitochondrial Protein Extraction Kit (Sangon Biotech, Shanghai, China), with some modifications. The resulting homogenate was then centrifuged at 3000 rpm for 10 min at 4°C. Next, the deposit was discarded and the supernatant was collected and again centrifuged at 12000 rpm for 30 min at 4°C. After that, the supernatant (cytosolic protein) was collected and centrifuged again (15000 rpm for 30 min at 4°C) to remove any microsomal contamination. The supernatant containing cytosolic protein with more purity was obtained; 0.1 ml Cytoplasmic Extract Buffer (supplemented with 0.1 μl protease inhibitor, 0.5 μl phosphatase inhibitor, and 0.1 μl DTT) was added to the deposit, followed by centrifugation at 13000 rpm for 10 min at 4°C. Then the supernatant was discarded and 0.1 ml Mitochondria Solvent Buffer (supplemented with 0.1 μl protease inhibitor, 0.5 μl phosphatase inhibitor, and 0.1 μl DTT) was added to the deposit, followed by centrifugation at 13000 rpm for 10 min at 4°C. The resulting supernatant was mitochondrial protein. The separated cytoplasmic and mitochondrial protein was subjected to Western blot analysis. We used cytochrome *c* oxidase subunit IV (COXIV) as subcellular marker for mitochondrial cytochrome *c* and GAPDH for cytosolic cytochrome *c*.

### Western blot

Rats were killed and then their stomachs were cut along the greater curvature. Briefly, specimens from the pyloric area were extracted in lysis buffer, homogenized, and centrifuged at 12000 × ***g*** at 26750 rpm for 5 min. Mitochondria were isolated when needed. Total proteins were examined by a BCA kit (Beyotime, China). Subsequently, the equal proteins were separated by SDS/PAGE (10% gels) and transferred to PVDF membrane (Millipore, U.S.A.). After blocking with 5% fat-free milk, primary antibodies against caspases (-3, -8, and -9), cytochrome *c*, Fas, Bax, Bid, NF-κB p65, Ki67, or p53 (all purchased from Santa Cruz, California, U.S.A.) were added, followed by incubations with secondary antibodies horseradish peroxidase-conjugated IgG. GAPDH served as the loading control. As for NF-κB nuclear p65, Lamin B served as the loading control. Bands were visualized with chemiluminescence kit (Applygen Technologies, China) and quantitated using NIH ImageJ software.

### Statistical analysis

Statistical analysis was performed with SPSS 17.0 (SPSS Inc, U.S.A.). Data were expressed as mean ± S.D. Differences between two groups were evaluated by Student’s *t* test. Differences between various groups were evaluated by one-way ANOVA with post-hoc test. Values of *P*<0.05 were considered statistically significant.

## Results

### XZ-1 decreased the incidence of GED

**Figure 1:**
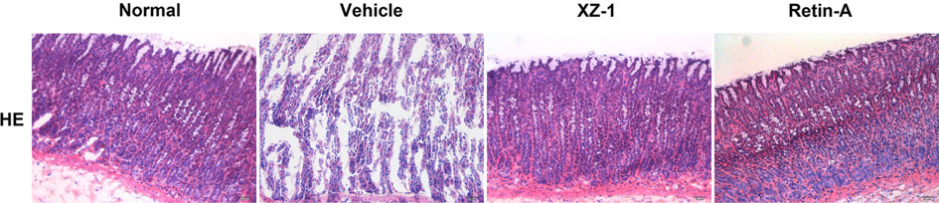
H&E staining of stomach in the four groups Rats were killed and then their stomachs were cut along the greater curvature. Paraffin sections were prepared from the pyloric area and subjected to H&E staining (scale bar: 20 μm). The vehicle group: the gastric mucosa dysplasia was severe, the gland structure was irregularly arranged, and the pathological mitotic phenomenon existed. The XZ-1 and Retin-A group: unobvious gastric mucosal gland hyperplasia and mild inflammation were observed. *n*=15 per group.

Rats were killed and then their stomachs were cut along the greater curvature. Paraffin sections were prepared from the pyloric area and subjected to H&E staining for routine pathological examination. As shown in Figure 1, the gastric mucosa dysplasia was severe, the gland structure was irregularly arranged, and the pathological mitotic phenomenon was observed in the vehicle group. After treatment with XZ-1 or Retin-A, the pathological changes of the pyloric area were almost comparable with that in the normal group, as evidenced by unobvious gastric mucosal gland hyperplasia and mild inflammation.

In addition, the incidence of GED in the XZ-1 (46.7%) group was significantly lower than that in the vehicle group (80%) (*P*<0.05, Table 1). Furthermore, the inhibitory effect of XZ-1 on the GED incidence was similar to Retin-A (53.3%).

**Table 1:** Incidence of GED

Group	*n*	Moderate dysplasia	Severe dysplasia	Incidence of dysplasia
Normal	15	0	0	0.00%
Vehicle	15	4	8	80.0%
XZ-1	15	3	4	46.7%*
Retin-A	15	4	4	53.3%

Rats in the normal, vehicle, XZ-1, and Retin-A group received treatment as described in ‘Materials and methods’ section.

* *P*<0.05 compared with Vehicle group.

### XZ-1 promoted cellular apoptosis of the pyloric area of the stomachs from GED rats

**Figure 2:**
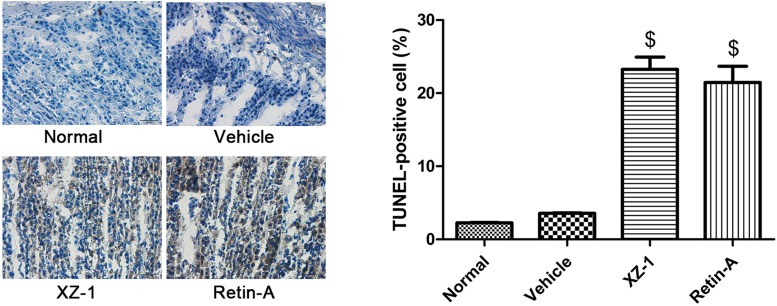
XZ-1 promoted cellular apoptosis of the pyloric area of the stomachs from GED rats Cell apoptosis in pyloric area of rats in the normal, vehicle, XZ-1, and Retin-A groups was evaluated by TUNEL staining. Left: representative images of TUNEL staining in the pyloric area of the stomachs from rats in the four groups. Right: the percentage of TUNEL-positive (brown) cells was shown. ^$^*P*<0.05 compared with Vehicle group. *n*=15 per group.

Specimens from the pyloric area were then subjected to TUNEL staining. Treatment with XZ-1 or Retin-A significantly up-regulated the percentage of TUNEL-positive cells in the pyloric area of the stomachs from rats compared with the vehicle group (*P*<0.05, Figure 2). The finding indicated that XZ-1, similar to Retin-A, promoted the cellular apoptosis of the stomach pyloric area from GED model rats.

### Expression of cleaved caspases (-3, -8, and -9) and cytochrome *c*

**Figure 3:**
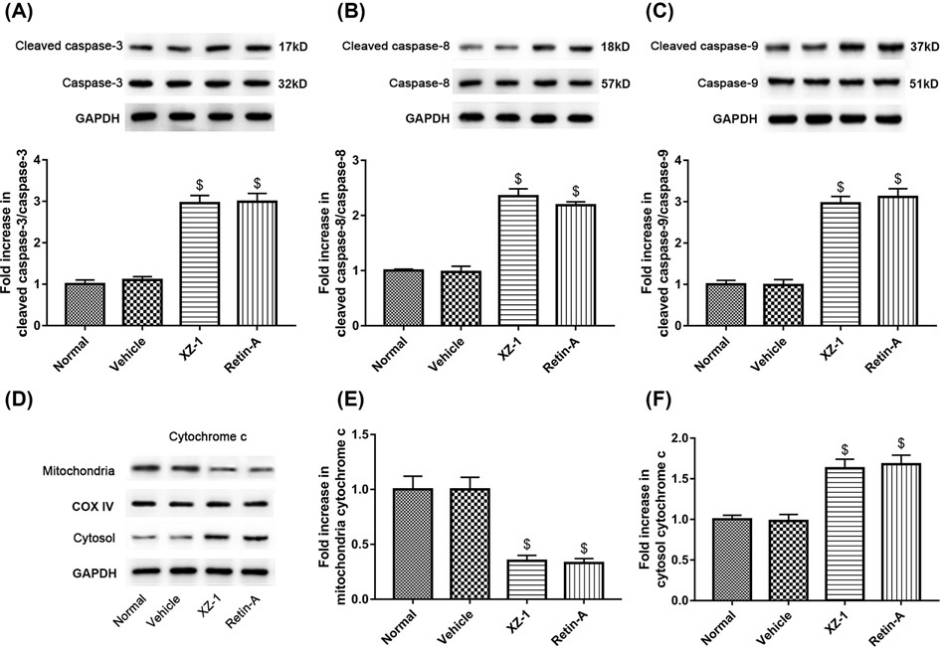
Cleaved caspases (-3, -8, and -9) and cytochrome *c* expression in the pyloric area of the stomachs from GED rats The protein expression of (**A**) cleaved caspase-3 and caspase-3, (**B**) cleaved caspase-8 and caspase-8, and (**C**) cleaved caspase-9 and caspase-9 in the pyloric area of the stomachs from rats in each group was evaluated by Western blot. GAPDH served as the loading control. The quantitative analysis of their blots was shown below. (**D**) The separated cytoplasmic and mitochondrial protein isolated from total proteins in the pyloric area of the stomachs from rats in each group was subjected to Western blot analysis of cytochrome *c*. COXIV served as the subcellular marker for mitochondrial cytochrome *c* and GAPDH for cytosolic cytochrome *c*. (**E**) The quantitative analysis of protein expression of mitochondrial cytochrome *c* was shown. (**F**) The quantitative analysis of protein expression of cytosolic cytochrome c was shown. ^$^*P*<0.05 compared with Vehicle group. *n*=15 per group.

To explore the protein expression of cellular apoptosis-related proteins, specimens from the pyloric area of the stomachs from rats in each group were homogenized, centrifuged, and then subjected to Western blot. Data revealed that the XZ-1 and the Retin-A group exhibited significantly higher protein expression of cleaved caspase-3, cleaved caspase-8, and cleaved caspase-9 compared with the vehicle group (*P*<0.05, Figure 3A–C). On the contrary, as shown in Figure 3D–F, the protein expression of mitochondrial cytochrome *c* in the XZ-1 and the Retin-A group was significantly lower than that in the vehicle group (*P*<0.05); however, the protein expression of cytochrome *c* in the cytosol showed the opposite expression pattern compared with the mitochondrial cytochrome *c* (*P*<0.05). Collectively, these results indicated that XZ-1, comparable with Retin-A, enhanced the protein expression of proapoptotic cleaved caspases (-3, -8, and -9) and facilitated the release of cytochrome *c* from mitochondria to cytosol.

### XZ-1 enhanced the protein expression of Fas, Bax, and Bid

**Figure 4:**
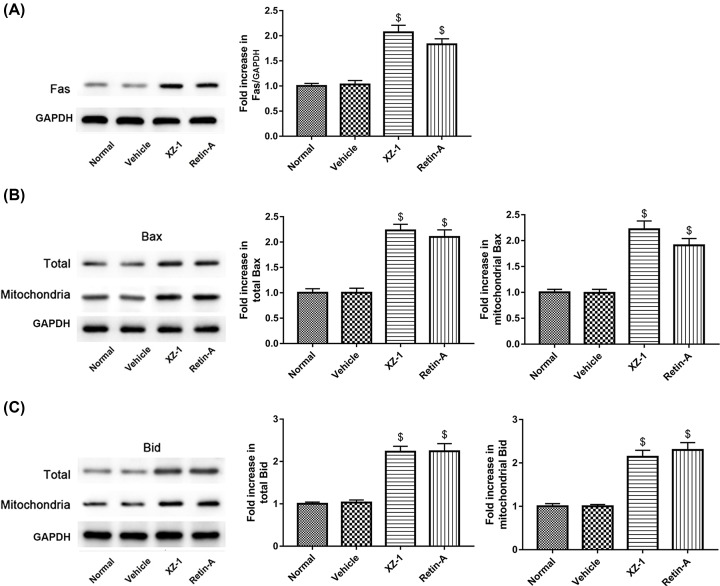
XZ-1 activated the expression of Fas, Bax, and Bid in the pyloric area of the stomachs from GED rats The protein expression of (**A**) Fas, (**B**) total and mitochondrial Bax, and (**C**) total and mitochondrial Bid in the pyloric area of the stomachs from rats in each group was evaluated by Western blot. GAPDH served as the loading control. The quantitative analysis of their blots was shown in the right. ^$^*P*<0.05 compared with Vehicle group. *n*=15 per group.

Next, we examined the effect of XZ-1 on other proapoptotic proteins including Fas, Bax, and Bid in the pyloric area of the stomachs from GED model rats. The protein expression of Fas in the XZ-1 and the Retin-A group was significantly higher than that in the vehicle group (*P*<0.05, Figure 4A). Likewise, both the XZ-1 and the Retin-A group also exhibited significantly higher protein expression of both total and mitochondrial Bax in comparison with the vehicle group (*P*<0.05, Figure 4B). Similar to the expression pattern of Bax, the protein expression of total Bid and mitochondrial Bid in the XZ-1 and the Retin-A group was also significantly higher than that in the vehicle group (*P*<0.05, Figure 4C).

### XZ-1 up-regulated the protein expression of NF-κB p65, Ki67, and p53

**Figure 5:**
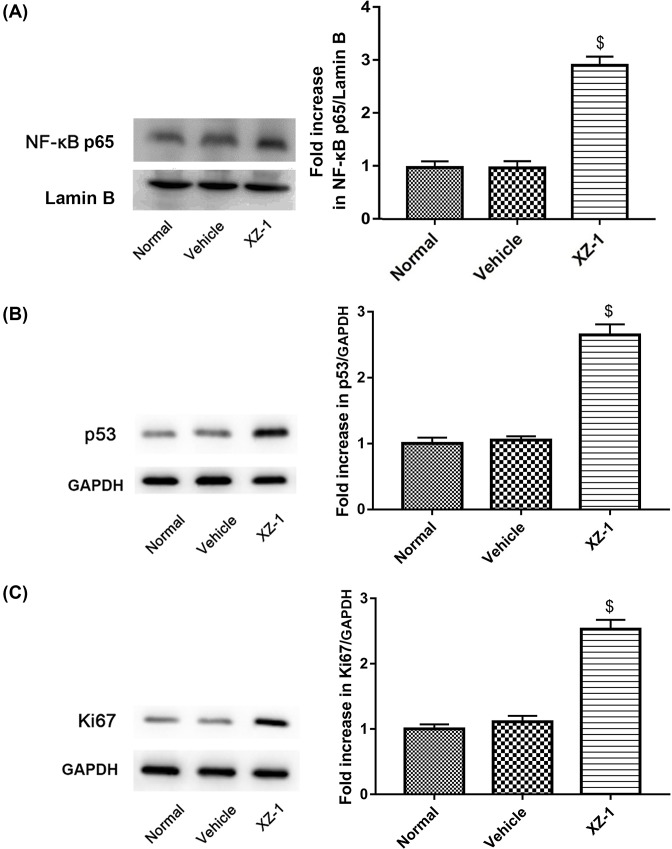
XZ-1 up-regulated the protein expression of NF-κB p65, p53, and Ki67 in the pyloric area of the stomachs from GED rats The protein expression of (**A**) NF-κB nuclear p65, (**B**) p53, (**C**) and Ki67 in the pyloric area of the stomachs from rats in each group was evaluated by Western blot. As for NF-κB nuclear p65, lamin B served as the loading control. As for p53 and Ki67, GAPDH served as the loading control. The quantitative analysis of their blots was shown on the right. ^$^*P*<0.05 compared with Vehicle group. *n*=15 per group.

To explore whether the NF-κB/Ki67/p53 pathway was involved in the XZ-1-induced gastric epithelial apoptosis of GED rats, we examined the effect of XZ-1 on protein expression of NF-κB nuclear p65, Ki67, and p53. Data revealed that the protein expression of NF-κB p65 in the pyloric area of the stomachs from rats in the XZ-1 group was significantly higher than that in the vehicle group (*P*<0.05, Figure 5A). For the protein expression of Ki67 and p53, similar results were observed (*P*<0.05, Figure 5B,C).

### Inhibition of NF-κB pathway suppressed p53 and Ki67, whereas inhibition of p53 pathway suppressed Ki67

**Figure 6:**
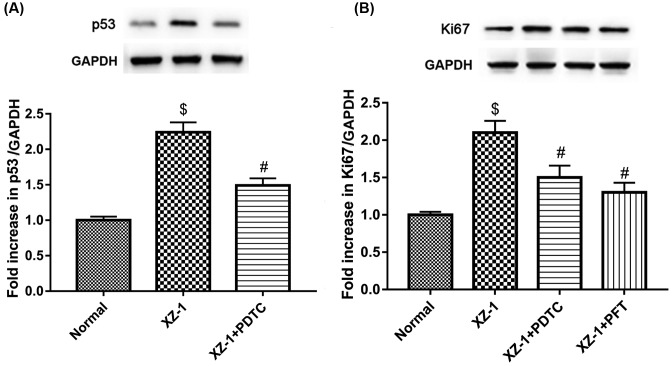
Inhibition of NF-κB suppressed the XZ-1-induced p53 and Ki67, and inhibition of p53 suppressed the XZ-1-induced Ki67 (**A**) Inhibition of NF-κB by PDTC significantly suppressed the XZ-1-mediated up-regulation of p53 protein expression. (**B**) Inhibition of NF-κB (by PDTC) or p53 pathway (by PFT) significantly suppressed the XZ-1-mediated up-regulation of Ki67 protein expression. GAPDH served as the loading control. ^$^*P*<0.05 compared with Vehicle group. ^#^*P*<0.05 compared with XZ-1 group. *n*=15 per group.

As shown in Figure 6A, the protein expression of p53 in the pyloric area of the stomachs from the rats in the XZ-1 group was significantly higher than that in the vehicle group (*P*<0.05); however, inhibition of NF-κB pathway by PDTC significantly down-regulated the XZ-1-induced protein expression of p53 (*P*<0.05). Furthermore, as shown in Figure 6B, similar results were observed in the protein expression of Ki67 (*P*<0.05). In addition, inhibition of p53 pathway by PFT also significantly suppressed the XZ-1-induced Ki67 protein expression (*P*<0.05). Our findings demonstrated that p53 could be suppressed by inhibition of NF-κB pathway, whereas Ki67 could be suppressed by inhibition of NF-κB or p53 pathway. These results provided an evidence of the correlation amongst NF-κB, p53, and Ki67 pathway.

### Inhibition of NF-κB or p53 attenuated XZ-1-induced cellular apoptosis of the pyloric area of the stomachs from GED rats

**Figure 7:**
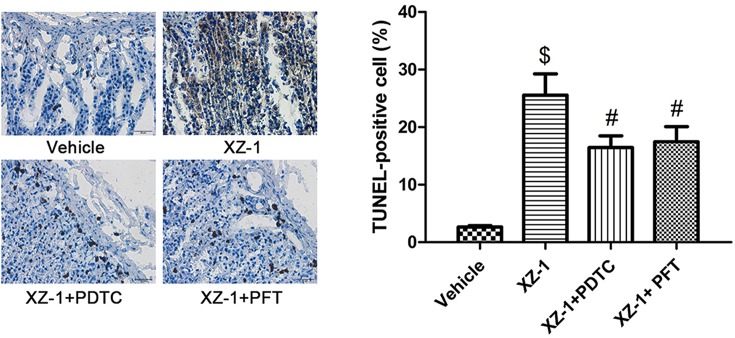
Inhibition of NF-κB or p53 attenuated XZ-1-induced cellular apoptosis of the pyloric area of the stomachs from GED rats Cell apoptosis in pyloric area of rats in the vehicle, XZ-1, XZ-1 + PDTC, and XZ-1 + PFT groups was evaluated by TUNEL staining. Left: representative images of TUNEL staining in the pyloric area of the stomachs from rats in the four groups. Right: the percentage of TUNEL-positive (brown) cells was shown. ^$^*P*<0.05 compared with Vehicle group. ^#^*P*<0.05 compared with XZ-1 group. *n*=15 per group.

Based on these findings, TUNEL staining was performed to further investigate whether the NF-κB/p53/Ki67 signaling pathway was involved in the XZ-1-induced cellular apoptosis of the pyloric area of the stomachs from GED rats. As shown in Figure 7, the cellular apoptosis in the pyloric area of the stomachs from rats in the XZ-1 group was significantly higher than that in the vehicle group (*P*<0.05); however, the percentage of TUNEL-positive cells in the pyloric area of the stomachs from rats in the XZ-1 + PDTC or XZ-1 + PFT group was significantly lower than that in the XZ-1 group (*P*<0.05). Our results suggested that the XZ-1-induced cellular apoptosis in the pyloric area of the stomachs from GED rats was attenuated by inhibition of p53 or NF-κB pathway.

### Inhibition of NF-κB or p53 pathway attenuated the XZ-1-mediated decline of GED incidence

Results of routine pathological examinations showed that the GED incidence in the XZ-1 + PDTC group (60.0%) and the XZ-1 + PFT group (66.7%) was significantly higher than that in the XZ-1 group (40%) (*P*<0.05, Table 2). Our results indicated that the XZ-mediated decline of GED incidence was attenuated by inhibition of NF-κB or p53 pathway.

**Table 2:** Incidence of GED

Group	*n*	Moderate dysplasia	Severe dysplasia	Incidence of dysplasia
Vehicle	15	5	8	86.7%
XZ-1	15	3	3	40.0%
XZ-1 + PDTC	15	3	6	60.0%*
XZ-1 + PFT	15	4	6	66.7%*

Rats in the vehicle, XZ-1, XZ-1 + PDTC, and XZ-1 + PFT group received treatment as described in ‘Materials and methods’ section.

* *P*<0.05 compared with XZ-1 group.

## Discussion

Carcinomatous evolution of GED increases proportionally with its histological grade [15]. Conventional treatments, including gastric acid suppression, gastric mucosa protection, and *Helicobacter pylori* eradication therapy, were commonly used to relieve symptoms and to improve inflammation of gastric mucus; while surgery or local endoscopic treatment was for high grade GED [11,16]. However, there have been almost no confirmed effective drugs to reverse GED so far.

The homeostasis of gastric mucosal epithelial cells is maintained by the balance between apoptosis of damaged or senescent cells and the proliferation of normal gastric epithelial cells [10]. Impairment of apoptosis was observed in intestinal metaplasia, GED, and gastric cancer [17,18]. Therefore, dysregulation of apoptosis might be one of the main causes of gastric cancer development.

Apoptosis is characterized by both morphological changes and activation of caspases [19,20]. Caspases, the central components in the apoptotic response [21], are expressed as inactive proenzymes and activated by proteolytic processing at internal aspartate residues when stimulated by an apoptosis-inducing signal [22]. Amongst these, caspase-3 is a key factor in apoptosis execution, whereas caspase-8 and caspase-9 are apoptosis activators [12]. Besides caspases, there are also several regulatory genes such as cytochrome *c*, Fas, Bax, Bak, and Bid which modulate cellular apoptosis. Fas ligand can induce apoptosis through binding to its cognate receptor Fas [20], the abnormality of which was previously reported to be correlated with dysplasia [23]. The release of cytochrome *c* from mitochondria to cytoplasm is regulated by anti-apoptotic (i.e. Bcl-2, Bcl-xL) and proapoptotic proteins (i.e. Bax, Bid, Bak) in the outer membrane of mitochondria [24]. Apoptosis occurs through either the intrinsic mitochondrial or extrinsic death receptor pathway [25]. In the former pathway, intracellular signals, including DNA damage and endoplasmic reticulum stress, target mitochondria either directly or through transduction by proapoptotic members of the Bcl-2 family (i.e. Bax, Bak) to induce mitochondrial membrane permeabilization (MMP). The mitochondria then release apoptogenic proteins (i.e. cytochrome *c*), consequently leading to caspase activation and apoptosis. While in the extrinsic pathway, the ligand-induced activation of death receptors, on the cytoplasmic side of the plasma membrane, stimulates the death-inducing signaling complex. This activates initiator caspase-8, triggering the activation of caspases to orchestrate apoptosis. Bid, activated by caspase-8, induces MMP and mediates the main cross-talk between the extrinsic and intrinsic pathways [25,26].

In our present study, we found that XZ-1 treatment decreased GED incidence and enhanced gastric epithelial apoptosis. Besides, XZ-1 up-regulated the protein expression of cleaved caspases (-3, -8, and -9), Fas, Bax, and Bid in the pyloric area of the stomachs from GED model rats. Moreover, XZ-1 facilitated the release of cytochrome *c* from mitochondria to cytosol. Mechanically, our results indicated that the increase in gastric epithelial apoptosis after treatment with XZ-1 may be due to the activation of the proapoptotic proteins including Fas, Bax, and Bid. Taken together, our findings indicated that the enhancement of apoptosis mediated by XZ-1 involved activation of apoptotic signaling pathway.

Ki67 is a large nucleolar phosphoprotein and closely correlates with the cell cycle [27]. The increase in NF-κB is related to a decrease in estrogen or progesterone receptors and increase in p53 accumulation [28]. The effect of NF-κB on cellular apoptosis relies on many factors such as inducer type, cell type, and others [28]. p53 and NF-κB inhibit the stimulation of gene expression by the other [28]. p53 can regulate the localization, expression, and activity of critical apoptotic effectors [29]. Abnormal expression of p53 was observed in gastric cancer and represents as a late event in the aggravation from the benign to the malignant phenotypes [30]. p53 activation can induce apoptosis and overexpressed p53 enhances levels of cell-surface Fas by promoting its trafficking from Golgi complex [31]. p53 exerts its proapoptotic effect by transcription-dependent and -independent manner [32]. p53 transactivation targets various molecules, such as Bcl-2 family (i.e. Bax, Bid), the apoptotic effectors (i.e. caspase-6, caspase-8), cell death receptors, cell death ligands, and others [33]. p53 can also induce the release of cytochrome *c* by stimulating the expression of the OKL38 tumour suppressor gene [34] and induce the expression of Fas and Fas ligand, the induction of which can augment the apoptotic signaling [35,36]. Moreover, p53 is also reported to activate Bid [37]. Our study demonstrated that XZ-1 treatment up-regulated protein expression of NF-κB p65, Ki67, and p53, indicating the involvement of NF-κB/Ki67/p53 pathway in the XZ-1-induced gastric epithelial apoptosis of GED rats. Additionally, we found that the XZ-1-induced p53 was suppressed by inhibition of NF-κB pathway. Furthermore, the XZ-1-induced Ki67 was suppressed by inhibition of NF-κB or p53 pathway. Moreover, we found that inhibition of NF-κB or p53 pathway attenuated the XZ-1-mediated induction of gastric epithelial apoptosis and the decline of GED incidence. Taken together, we concluded that XZ-1 activated cell apoptosis of GED via NF-κB/p53/Ki67 signaling pathway. This might be one of the mechanisms by which XZ-1 reversed GED.

In summary, our results demonstrated that XZ-1 dramatically decreased the GED incidence and enhanced gastric epithelial apoptosis, almost equivalent effect exerted by Retin-A. Besides, XZ-1 activated apoptosis signaling pathway, which was related with NF-κB/p53/Ki67 signaling. Our study provides a new explanation of the treatment of GED by XZ-1.

## Abbreviations

Bax: Bcl-2-associated X protein
Bcl-2: B-cell lymphoma 2
Bcl-xL: B-cell lymphoma-extra large
Bid: BH3 interacting domain death agonist
Caspase: cysteine-dependent aspartate-specific protease
Fas: Fas cell surface death receptor
GAPDH: glyceraldehyde 3-phosphate dehydrogenase
GED: gastric epithelial dysplasia
H&E: Hematoxylin and Eosin
IG: intragastric
Ki-67: proliferation marker protein Ki-67
MMP: mitochondrial membrane permeabilization
MNNG: N-methyl-N-nitro-N-nitrosoguanidine
NF-κB: nulclear factor-kappaB
OKL38: homo sapiens pregnancy-induced growth inhibitor
PDTC: pyrrolidinedithiocarbamate
PFT: pifithrin-α
p53: tumor protein p53
TUNEL: terminal deoxynucleotidyl transferase dUTP nick end labeling
XZ-1: Xiaozeng No. 1
